# Antidepressant-like effects of benzodiazepine site inverse agonists in the rat forced swim test

**DOI:** 10.1186/2050-6511-13-S1-A1

**Published:** 2012-09-17

**Authors:** Janko Samardžić, Dragan I Obradović

**Affiliations:** 1 Institute of Pharmacology, Clinical Pharmacology and Toxicology, Medical Faculty, University of Belgrade, 11129 Belgrade, Serbia

## Background

There are three kinds of allosteric modulators acting through the benzodiazepine (BZ) binding site of the GABA_A_ receptor: positive (agonist), neutral (antagonist), and negative (inverse agonist) modulators. Agonists and inverse agonists commonly exert bidirectional influences on observed behavioral parameters. In the present study we have investigated the modulation of behavioral responses to environmental novelty in two unconditioned paradigms: spontaneous locomotor activity (SLA) and forced swim test (FST), elicited by DMCM (methyl-6,7-dimethoxy-4-ethyl-beta-carboline-3-carboxylate), a non-selective inverse agonist, in the dose range that previously did not produce anxiogenic effects and convulsions.

## Methods

SLA in the test cage (40 x 25 x 35 cm) during 30 min was recorded automatically, beginning 20 min after i.p. injections (DMCM 0.1, 0.5 and 1 mg/kg) without any habituation. FST was performed in a glass cylinder, 45 cm high, 20 cm diameter filled with water up to a height of 20 cm, with a temperature of 21–23 °C. Male Wistar rats were exposed to two swimming sessions (an initial 15-min pretest session, followed 24 h later by a 5-min test session). The animals received i.p. 0.1, 0.5 and 1 mg/kg of DMCM or vehicle, 20 min before the test session. A rat was considered immobile whenever it floated passively in the water and only made movements necessary to keep its head above the water line.

## Results

ANOVA showed a significant effect of treatment on the total immobility time of the animals during 30 min of monitoring of spontaneous locomotor activity (p < 0.05). Namely, Dunnett's analysis showed that the highest applied dose of DMCM (1 mg/kg) exerted the activity-decreasing effect related to vehicle. In FST during the test session, ANOVA indicated statistically significant effects of DMCM (p < 0.05) on the average immobility time of animals. Dunnett’s analysis showed that DMCM (1.0 mg/kg) significantly increased immoblity, but at the lowest applied dose DMCM (0.1 mg/kg) decreased immobility, and exerted acute antidepressant-like effects.

## Conclusions

These data suggest that negative modulation at GABA_A_ receptors might have triggered the acute antidepressant-like effects in rats and these effects were not confounded by locomotor influences. On the other hand, these effects are not straightforward, because they exert a kind of bimodal influence. Furthermore, these results encourage the synthesis of new BZ site ligands, aimed to possess more selective affinity/efficacy profiles.

